# Trends and predictors of unmet need for family planning among women living with HIV in Zambia: implications for elimination of vertical transmission of HIV

**DOI:** 10.1186/s12889-024-18127-3

**Published:** 2024-04-11

**Authors:** Edgar Arnold Lungu, Mwimba Chewe

**Affiliations:** 1 Health and HIV Section, UNICEF Zambia, PO Box 33610, Alick Nkhata Rd, Long Acres, Lusaka, Zambia; 2 Department of Public Health, University of Zambia, PO Box: 50110, Burma Road, Lusaka, Zambia

**Keywords:** HIV, Vertical transmission, Unmet need, Family planning, Women, Zambia, Integration

## Abstract

**Background:**

Prevention of vertical (mother to child) transmission of HIV is one of the key strategies towards HIV epidemic control. Despite considerable progress over the past decade in Zambia, the country is yet to reach global and national target for elimination of vertical transmission of HIV. Avoidance of unintended pregnancy among women living with HIV is one of the cost-effective interventions in a comprehensive approach to prevent vertical transmission of HIV. Therefore, this study aimed at ascertaining trends in and predictors of unmet need for family planning among women living with HIV in Zambia.

**Methods:**

The study employed a repeated cross sectional (RCS) study design, using data from the three (3) most recent consecutive rounds of the Zambia Demographic and Health Survey (ZDHS) conducted in 2007, 2013/2014 and 2018. The study used data from a total of 27,153 women aged 15–49 years over the three survey periods among whom 4,113 had an HIV positive result following a rigorous HIV testing algorithm of the demographic and health surveys, and these constituted our sample size of women living with HIV. We used descriptive statistics and logistic regression analyses to respectively ascertain trends in and predictors of unmet need for family planning among women living with HIV.

**Results:**

Over the three survey points, unmet need for family planning among women living with HIV has largely remained unchanged from 20.8% in 2007 to 20.5% in 2013/14 and 21.1% in 2018 DHS. Residence, age of women, household wealth, woman’s parity, employment, and age of spouse emerged as significant predictors of unmet need for family planning among women living with HIV in Zambia.

**Conclusion:**

Preventing HIV infection in a child preserves life, contributes to improving quality of life from its early stages and averts lifetime costs of HIV treatment and associated healthcare costs. There is need to consider optimization of interventions to prevent vertical transmission of HIV including shaping programming regarding preventing unintended pregnancies among women living with HIV. Among other aspects, policy and practice need to strengthen SRH/HIV integration and better target rural residents, younger women, those with high parity and consider positive male engagement to reduce unmet need for family planning among women living with HIV.

## Introduction

**Fig. 1:**
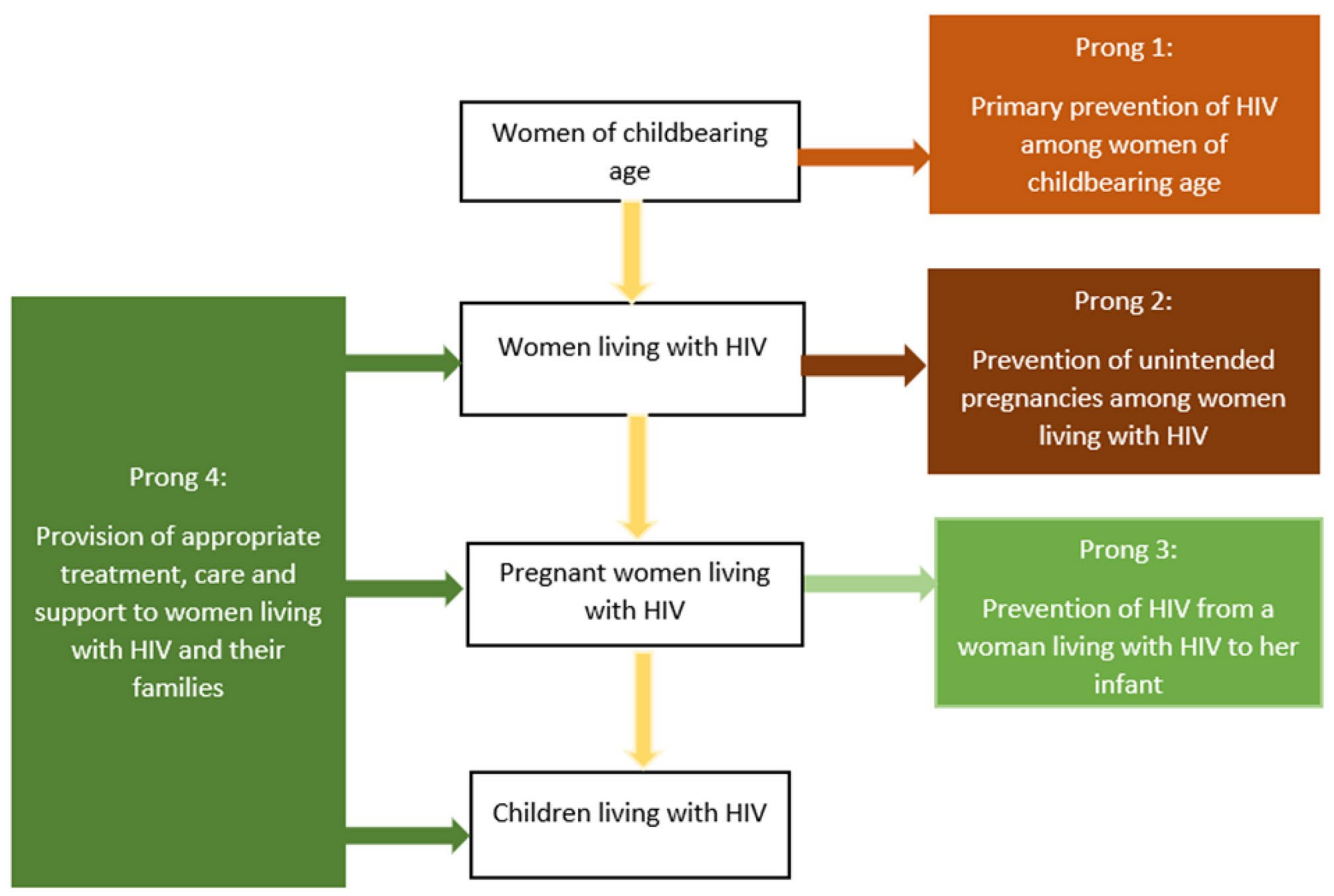
Four-pronged approach for comprehensive prevention of vertical transmission of HIV. Source: authors’ illustration based on presentation by Dr Lee Fairlie, South Africa HIV Society, 2014

Prevention of vertical (mother to child) transmission of HIV has been integral to global HIV prevention interventions since the late 1990’s following the success of the short-course Zidovudine and single-dose nevirapine clinical trials [1–3]. Subsequent recommendations from the World Health Organisation on the use of Anti-retroviral (ARV) drugs for prevention of vertical transmission of HIV which were first issued in 2000 [4] also provided prominence to programming. In the context of Millennium Development Goals (MDGs), a more comprehensive four-pronged approach to prevention of vertical transmission of HIV was launched by the United Nations in 2003 [5] and eventually adopted by many countries with high HIV burden. The four-pronged approach focuses on provision of cost-effective interventions at critical stages during the perinatal period to prevent vertical transmission of HIV and to improve maternal health (see Fig. 1).

The first prong focusses on primary prevention of HIV among the general population, especially among women of reproductive age, given that averting new HIV infections in this population group entails parents that are free of HIV, effectively eliminating the risk of vertical transmission. The second prong entails prevention of unwanted pregnancies among women living with HIV. Whilst more potent ARV drugs currently exist and significantly reduce probability of vertical transmission of HIV, avoidance of unintended pregnancy among women living with HIV provides a cost-effective way as it eliminates the risk of transmission. The third prong involves provision of HIV testing for case detection and linkage to sustained care and treatment especially provision of ARVs to pregnant and breastfeeding women who are living with HIV to reduce probability of vertical transmission. The fourth prong requires provision of care, treatment and support services to women living with HIV, their partners, their children, and families with view to provide HIV prophylaxis for HIV exposed children, continued care, and treatment for women to reduce new HIV infections in the breastfeeding period [5].

Although all four prongs are central to preventing vertical transmission of HIV and promoting the health and wellbeing of women, programmes in many countries have predominantly focused on identifying pregnant women living with HIV and initiating them on treatment (prong 3) [6]. Essentially, prong 3 and 4 interventions have been synonymous to prevention of vertical transmission of HIV in many high HIV burden countries. Evidence on effectiveness of Option B + and Treatment as Prevention (TasP) have arguably shaped the context for programmes to prevent vertical transmission of HIV [7] to date.

From 2015, UNAIDS initiated an advocacy campaign termed “a quarter for prevention” which called on countries to increase investment in HIV prevention by allocating 25 percent of resources in HIV and AIDS response to HIV prevention interventions [8]. This campaign, aimed at revitalising HIV prevention, would have predominantly served prong 1, given that “we cannot treat ourselves out of the HIV epidemic” [9]. Whereas in theory, this made investment sense, actual implementation still principally focused on care and treatment interventions (mostly prongs 3 and 4). Granted this programming context, it is probable that the success, such as averting more than 110,000 new HIV infections in children between 2012 and 2021 in Zambia [10] and a 38 per cent reduction in vertical transmission of HIV between 2009 and 2014 [11] has largely been due to increasing coverage of prong 3 and 4 interventions.

Despite the prominence of prongs 3 and 4 in programmes aimed at reducing vertical transmission in high HIV burden countries including Zambia, evidence exists suggesting the centrality of prong 2 (prevention of unintended pregnancies among women living with HIV). For example, a study in PEPFAR supported countries estimated that annual number of unintended HIV-positive births averted by contraceptive use ranged from 178 in Guyana to over 120,000 in South Africa with minimum annual cost savings to prevent unwanted HIV-positive births reaching over 2.2 million United States Dollars in South Africa [12]. There is also evidence that even modest decreases in the number of pregnancies to women living with HIV could significantly reduce new HIV infections among children from both women living with HIV who are not on treatment (where prong 2 has the largest effect) and women living with HIV who are on treatment and desire to space or limit birthing [13]. Moreover, some regional evidence suggests that an estimated “333,000 new infant infections could be averted annually, if all women in the sub-Sahara Africa who did not wish to become pregnant could have access to contraceptive services” [14]. Furthermore, mathematical modelling based on data from Uganda predicted that the effect of preventing unintended pregnancies in HIV positive women could contribute to averting vertical transmission of HIV equally or more than what is achieved through the use of ART [13]. Noticeably, much of this evidence was in the pre-Option B plus and universal ART eligibility programming era (i.e. where ART was only given to the mother for up to 6 weeks and later up to two years postpartum for purposes of preventing vertical transmission of HIV).

Whereas more recent estimates, in the context of high ART coverage among pregnant women show lower numbers of infant HIV infections averted by contraception than previously estimated, the overarching conclusion from studies remains that contraception plays a significant role in preventing new infant HIV infections [14]. Moreover, beyond the significant role in preventing vertical transmission of HIV, it is worthwhile to note that family planning services are an essential health care service with important implications for women’s health, wellbeing, and autonomy. Indeed, the overarching goals of programmes to prevent vertical transmission of HIV are twofold.; (i) eliminate vertical transmission of HIV and to (ii) improve women’s health [11]. On this basis, investing in prong 2 has significant merit to HIV epidemic control, especially the elimination of vertical transmission of HIV agenda, and overall women’s health. Arguably, the optimal benefits of investing in prong 2 have not been reaped [14]. Expanding family planning services for women living with HIV presents opportunities to substantially contribute towards eliminating vertical transmission of HIV in high HIV burden countries such as Zambia.

Though not extensively studied, literature largely suggests that determinants of contraceptive uptake and unmet need for family planning are similar for women irrespective of the HIV sero-status. Studies have predominantly identified geographical residence, age of women, socioeconomic status, education, partners age, partners education as common determinants of unmet need for family planning [15–17]. The direction of association is mainly that worse off groups (poorer, less educated, rural residents, limited autonomy) have lesser odds of use of family planning and conversely higher odds of unmet need for family planning [15–17].

Zambia adopted and developed plans to pursue the global agenda for elimination of vertical transmission of HIV with two impact targets, namely, a vertical transmission rate of HIV of less than 5% in breastfeeding population; and a population case rate of new pediatric HIV infections due to vertical transmission of equal to or less than 50 cases per 100,000 live births, [18–19]. Whilst significant progress has been made, these targets were not achieved by 2021. Indeed, Zambia has sustained high coverage of HIV testing for pregnant women attending antenatal care which currently stands at 95% and consequently a high Anti-Retroviral Therapy (ART) coverage among pregnant women living with HIV, currently standing at 97% in 2021 [10]. Nonetheless, rate of vertical transmission of HIV at the end of exposure period (at the end of breast feeding) remains high at 8% falling short of the targets for eliminating vertical transmission of HIV. This reflects the need to re-examine current implementation including sustaining gains in prongs 3 and 4 while strengthening evidence and investment in prongs 1 and 2 which have hitherto been of relative low worth. Indeed, achieving the agenda for elimination of vertical transmission of HIV requires comprehensive programming espousing optimal implementation of the four-pronged approach.

In the most recent Demographic and Health Survey (DHS) report, unmet need for family planning is estimated to be 19.7% in Zambia [20]. There is paucity of consistent nationally representative data on unmet need for family planning among women living with HIV, with few studies reporting this statistic at a point in time (cross-sectional). In some cases, programme level data suggest an increasing uptake of family planning among women living with HIV as part of projects and mostly within the project’s lifespan [21], with no or little evidence of national level impact. While there is some literature on unmet need for family planning among women in Zambia, there is still a gap in understanding trends in unmet need for family planning amongst women living with HIV. Specifically, questions such as “Has the unmet need for family planning among women living with HIV changed over time?”; “What factors predict unmet need for family planning among women living with HIV in Zambia?” are yet to be comprehensively answered. Our study seeks to contribute to evidence in this regard and explore programmatic implications on the path to elimination of vertical transmission of HIV in Zambia - a global priority country for HIV epidemic control and one of the first-tier countries for the Global alliance on ending AIDS in children by 2030.

## Methodology

### Study design and data sources

We undertook a secondary analysis of data from Demographic and Health Surveys (DHS) conducted in Zambia to understand trends and determinants of unmet need for family planning among women living with HIV. We adopted a repeated cross sectional (RCS) study design, using data from the three [3] most recent consecutive rounds of the Zambia Demographic and Health Survey (ZDHS). The RCS data structure is particularly beneficial because it introduces a dynamic component to the study of cross-sectional units and allows for the exploration of time-varying relationships which could broaden insight into the relationship being investigated [22].

Demographic and Health Surveys (DHS) are nationally representative population-based surveys conducted by the ICF Macro in collaboration with governments in about 90 countries, typically every four to five years. The DHS provide data on various health and demographic indicators including mortality, sexual and reproductive health, HIV, health status and health seeking, and child nutrition. In Zambia, five DHS have been conducted in 1992, 1996, 2001/02, 2007, 2013/2014 and 2018 [20].

Our study used DHS data from surveys conducted in 2007, 2014 and 2018. These surveys used the same sampling strategy, defined the outcome variable (unmet need for family planning) and other variables of interest (such as HIV and all demographic variables) in the same way and had robust HIV testing algorithm with highest diagnostic accuracy based on prevailing technology in respective years, making the data comparable over the three surveys. The DHS use robust quality assurance measures throughout the data collection and management process, the findings are highly regarded and used to inform policy and practice. We accessed all relevant DHS data sets upon request through the ICF Macro data management portal as custodians of DHS data. For each survey, two sets of data were availed, namely women’s data file constituting all sociodemographic and sexual and reproductive health data including family planning, and the HIV data file that included data on HIV testing and results.

### Definition of variables

In DHS, unmet need for family planning is typically defined to encompass two main groups, namely: (i) unmet need for spacing which means fecund women of childbearing age who would want to space their birth intervals but are currently not on any modern contraceptive method and (ii) unmet need for limiting referring to fecund women of childbearing age who would desire no additional children and are presently not on any modern contraceptive method [23]. Essentially, questions focus on fertility intentions and current contraceptive use.

Our outcome variable (unmet need for family planning) definition was consistent with that of the DHS albeit recognising that some discourse has contested and cited various challenges with the indicator definition. Some salient issues and challenges raised include, that: “users of traditional methods are treated as non-users based on the implicit assumption that they lack access to or information concerning more effective alternatives; the concept of unmet need is based on the discrepancy between the future bearing wishes and contraceptive use rather than from a direct expression of need by respondents”, and that all estimates of unmet need are exclusively based on women’s reports [24]. This notwithstanding, information on unmet need for family planning generated by DHS still reflects an acceptable measure and has been useful in informing policy and practice for many years.

To ascertain the relationship between different individual characteristics and unmet need for family planning among women living with HIV, we included several demographic and socioeconomic variables. The socioeconomic indicators we adopted included highest level of education, religion, whether respondent has worked in the past 12 months and the eligible women’s wealth level proxied by a household wealth index. Demographic variables included age, partner’s age, and area of residence. Other variables included the total number of children ever born to a woman, partner’s education level, visit to health facility in the past year as well as whether the respondent is the primary decision maker on their health.

Table 1 provides definitions and respective measurements for key outcome and predictor variables as used in the DHS and applied in our study.

**Table 1:** Definitions and measurement of key variables

Variable	Variable definition	Measurement
Unmet need for family planning	Fecund women of reproductive age who did not desire to have children at the time of the survey but were not using any modern methods of contraception.	Combined measure of fertility intention (in this case not wanting to have a child either due to need for spacing children or did not need to have any more children) and use of contraceptive method (in this case not using contraceptive method).
Women living with HIV	Women living with the Human Immuno-deficiency virus (HIV) ascertained through a positive HIV blood test result conducted during the survey.	An HIV testing algorithm with high diagnostic accuracy was used during the surveys to ascertain HIV sero-status. Constituted women who had prior knowledge of HIV positive sero-status which was still ascertained through confirmatory test and women who did not have prior knowledge of their HIV sero-status and had an HIV positive test result.
Wealth index	Level of women’s household wealth.	Calculated and provided in DHS data set as standard household wealth measurements constructed using Principal Component Analysis (PCA) categorizing wealth into five levels of: poorest, poorer, middle, richer, and richest.
Year	The year during which the DHS was conducted.	Data sets provided by year of the survey where variables of interest were extracted.
Residence	Geographic entity where a woman was residing at the time of the survey classified as urban or rural.	DHS adopts country definition of what constitutes urban or rural.
Education	Education level attained by woman.	Highest level of formal education attained as reported by study participant.
Age	Age of woman (in years) at the time of survey data collection.	Age collected as a continuous variable and categorized into five-year age groups. DHS collects data from women aged 15 to 49 years.

### Study participants

Our study used a dataset constituting of data for 27,153 women aged 15–49 years over the three survey points, 2007, 2013/14 and 2018. Of these, 4,113 **(**16%) had an HIV positive test result and constituted the sample size for our study.

### Data synthesis and analysis

In the first stage of data synthesis, we merged two data sets (i.e. women’s HIV data set and women’s data set on all DHS parameters which include family planning data) from the ZDHS for the years 2007, 2013/14 and 2018. This was done using the unique identification codes for the survey participants to ensure that, although compiled separately, the two data sets were effectively linked to the extent that the family planning data and HIV data at individual level were indeed for the same woman. Expertise to undertake this was available from the authors and additional technical support, for purposes of quality assurance, was solicited from an expert working with the Central Statistics Office who has vast experience in working with DHS data.

**Fig. 2:**
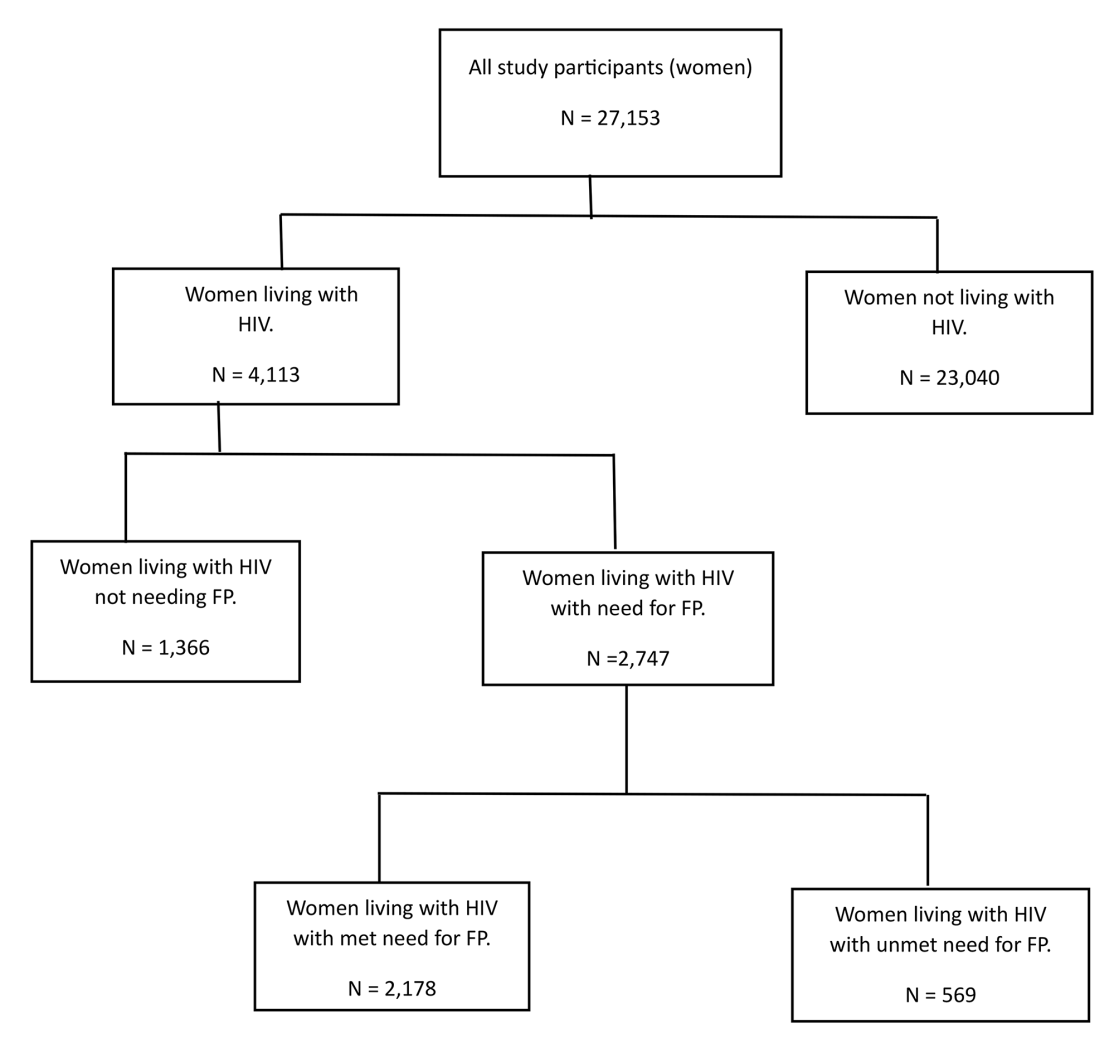
Data extraction steps for the study

Using the HIV test result data, we extracted all women who had a positive HIV test result as a subgroup of interest and were able to ascertain their status regarding family planning, specifically whether the need for family planning was met or unmet. Figure 2 illustrates the data extraction and filtering flow.

We calculated unmet need for family planning for women living with HIV using the standard DHS definition for each DHS database (see section on definition of variables). We then undertook trend analysis using DHS surveys as data time points to observe any changes in unmet need for family planning among women living with HIV over time.

Our analysis also used STATA command that ensures that survey weights are applied when estimating percentages. To determine the factors associated with unmet need for family planning amongst women living with HIV in Zambia, we adopted a logistic regression analysis. We used a backward stepwise regression modelling approach which allows inclusion of multiple variables (saturated model) and gradually eliminates variables at each step to arrive at a reduced model that best fits the data. This approach also reduces multicollinearity, is one way of resolving overfitting and has been used to good effect [25].

### Ethical review

We used secondary data from Demographic and Health Surveys undertaken in Zambia for which ethical approvals were granted by institutional review boards (IRBs) at ICF and the Tropical Diseases Research Centre (TDRC) in Zambia [20]. We obtained approval to access and use the data from the DHS Program.

## Results

### Sociodemographic characteristics

Our study included a 4,113 who had an HIV positive test result over the three surveys. The HIV results are based on robust HIV testing algorithm of the DHS. In respective DHS, the HIV prevalence ranged from 14% in 2018 to 16% in 2007.

Sociodemographic characteristics of our sample are as presented in Table 2. Overall, majority (93.9%) of participants had at least attained primary education (43.4%, 43.9% and 6.6% with primary, secondary, and tertiary education respectively). Women residing in urban areas constituted the majority (64.4%), women aged 25–39 years accounted for about 60% of the sample and slightly more than half (55%) were married. The mean age was 33 years (SD: 8.2, Range: 15–49) and that of partners to women included in the sample was 40 years (SD: 9.2, Range: 18–99). On average, women included in the study had about 3 children (SD: 3, Range: 0–11). The 2013/14 DHS period had the largest contribution to the overall sample (50.4%).

**Table 2:** Sociodemographic characteristics of participants

Variable	Women Living with HIV (*N* = 4,113)		Women Living with HIV with met need for FP (*N* = 2,178)		Women Living with HIV with unmet need for FP (*N* = 569)	
	n	Per cent (column %)	n	Per cent (Row %)	n	Percent (Row %)
**Residence**						
Urban	2,648	64.4%	1,402	80.4%	341	19.6%
Rural	1,465	35.6%	776	77.3%	228	22.7%
**Age**						
15–19	186	4.5%	50	67.6%	24	32.4%
20–24	572	13.9%	325	80.9%	77	19.2%
25–29	761	18.5%	481	82.1%	105	17.9%
30–34	840	20.4%	531	81.2%	123	18.8%
35–39	782	19.0%	424	79.6%	109	20.5%
40–44	622	15.1%	277	74.7%	94	25.3%
45–49	350	8.5%	90	70.9%	37	29.1%
**Education**						
No Education	251	6.1%	113	72.9%	42	27.1%
Primary	1,784	43.4%	933	76.1%	292	23.9%
Secondary	1,806	43.9%	981	82.3%	211	17.7%
Higher	272	6.6%	151	86.3%	151	13.7%
**Wealth Index**						
Poorest	393	9.6%	188	76.1%	59	23.9%
Poorer	515	12.5%	260	73.5%	94	26.5%
Middle	856	20.8%	451	77.0%	135	23.0%
Richer	1,212	29.5%	674	80.9%	159	19.0%
Richest	1,137	27.6%	605	83.2%	122	16.7%
**Marital Status**						
Single	606	14.7%	178	78.4%	49	21.6%
Married	2,258	54.9%	1,609	77.8%	459	22.2%
Living with partner	33	0.8%	26	81.2%	6	18.7%
Separated	171	4.2%	70	87.5%	10	12.5%
Widow	459	11.2%	91	87.5%	13	12.5%
Divorced	586	14.3%	32	86.4%	32	13.6%
**Religion**						
Catholic	699	17.0%	392	81.3%	90	18.7%
Protestant	3,357	81.7%	1,757	79.0%	466	21.0%
Muslim	21	0.5%	12	70.6%	5	29.4%
Other	34	0.8%	17	70.8%	7	29.2%
**Year**						
2007	451	11.0%	240	79.2%	63	20.8%
2013	2,071	50.4%	1,124	79.5%	289	20.5%
2018	1,591	38.7%	814	79.0%	217	21.1%
**Currently Working**						
No	1,697	41.3%	899	77.3%	264	22.7%
Yes	2,410	58.7%	1,275	80.6%	304	19.3%
	**Mean**	**SE**	**Mean**	**SE**	**Mean**	**SE**
**Partners Age**	39.9	0.19	36.6	0.08	38.6	0.13
**Children Ever Born**	3.2	0.04	3.5	0.02	3.7	0.03

Note: Data for overall demographic characteristics for women living with HIV is presented in column percentages and data on met and unmet need for family planning among women living with HIV are presented in row percentages

### Levels and trends of unmet need for family planning among women living with HIV

**Fig. 3:**
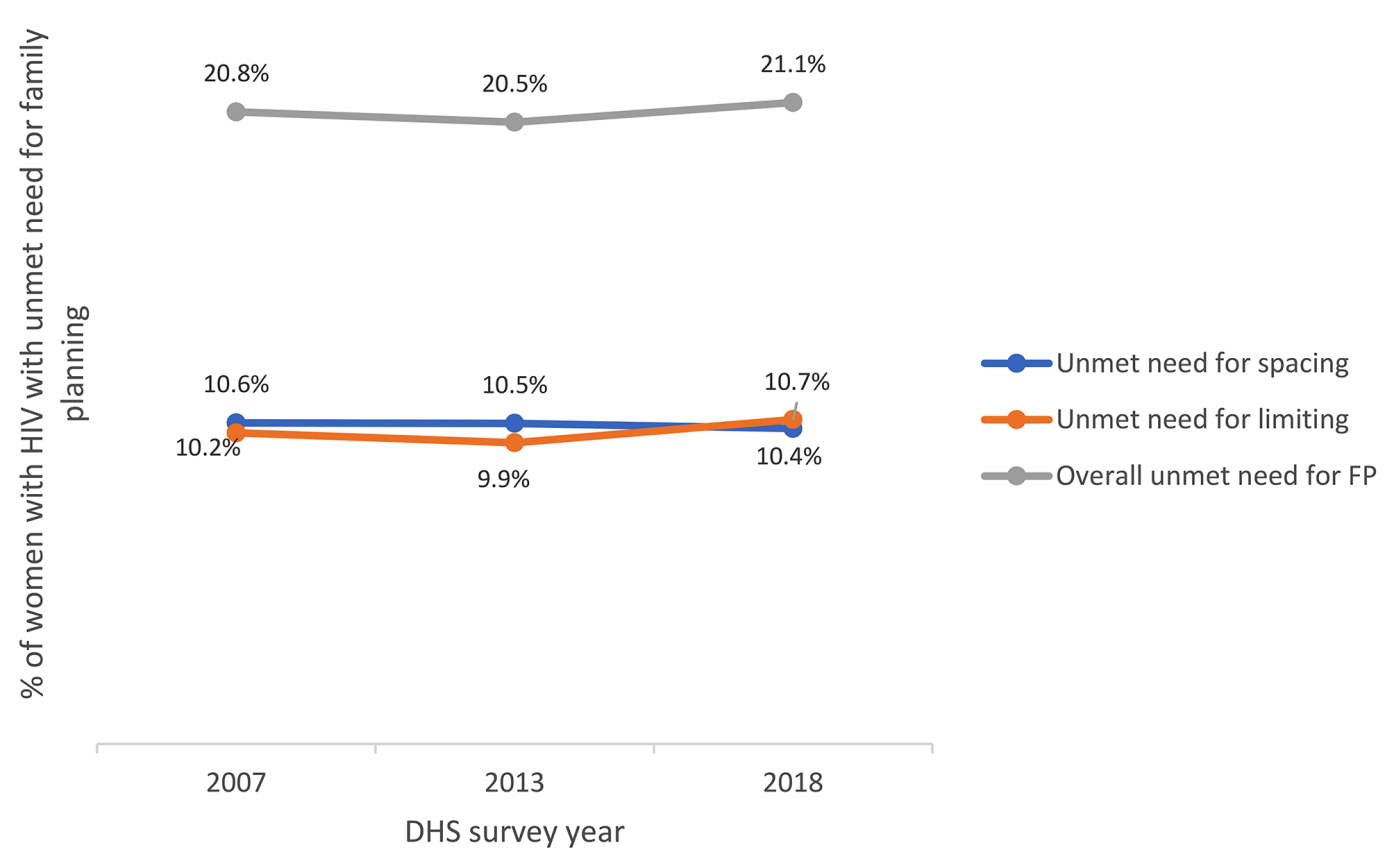
Proportion of women living with HIV with unmet need for spacing, unmet need for limiting and overall unmet need for family planning by year of survey

Results show that over the three survey points, unmet need for family planning among women living with HIV has largely remained unchanged from 20.8% in 2007 to 20.5% in 2013/14 and 21.1% in 2018 DHS (see Table 2; Fig. 3) with the small differences not being statistically significant (see Table 2). Further analysis, disaggregating unmet needs for spacing and limiting reveal that the two categories contribute relatively the same to the overall unmet need for family planning among women living with HIV with no survey point showing a difference of more than a percentage point (see Fig. 3).

### Factors associated with unmet need for family planning among women living with HIV

Table 3 presents results of our final model of multivariate logistic regression analysis for factors associated with unmet need for family planning among women living with HIV over the three Zambia DHS survey points. The odds of having an unmet need for family planning increased with an increasing number of children ever born (parity) to a woman at the point of the survey (AOR = 1.14, 95% CI: 1.07– 1.21). Similarly, unmet need for family planning among women living with HIV increased with an increasing age of spouse/partner (AOR = 1.02, 95% CI: 1.00–1.04).

Compared to women residing in rural areas, urban women are less likely to have unmet need for family planning (AOR = 0.615 95% CI: 0.458 0.8270). Age of women also emerged as a significant predictor of unmet need for family planning with women aged 20–49 years (Age 20–24: AOR = 0.38, 95% CI: 0.18–0.81; Age 25–29: AOR = 0.33, 95% CI: 0.15–0.69; Age 30–34: AOR = 0.32, 95% CI: 0.15–0.71; Age 35–39: AOR = 0.28, 95% CI: 0.12–0.64; Age 40–44: AOR = 0.32, 95% CI: 0.13–0.77; Age 45–49: AOR = 0.33, 95% CI: 0.12–0.89) having lesser odds of having an unmet need for family planning compared to adolescent girls aged 15–19. Women who were working at the time of the survey had lesser odds of having unmet need for family planning compared to women who reported not to be working (AOR = 0.78, 95% CI: 0.63–0.98). Whilst there was no significant difference in unmet need for family planning among women in the first two wealth quintiles (poorer and middle), compared to women in lowest wealth quintile (poorest), those in fourth (richer) (AOR = 0.53, 95% CI: 0.33–0.85) and fifth (richest) (AOR = 0.42, 95% CI: 0.23–0.72) wealth quintiles had lesser odds of having unmet need for family planning.

There was no significant difference in unmet need for family planning among women living with HIV by survey year, marital status, and women’s level of education. Additionally, factors such as, whether respondent visited health facility in 12 months preceding the survey, and decision maker on health care, that were included in the regression model, were not significantly associated with unmet need for family planning among women living with HIV.

**Table 3:** Multivariable logistic regression results of factors associated with unmet need for family planning among women living with HIV.

Dependent Variable: Unmet Need for Family Planning among women living with HIV	Logistic Regression with Adjusted Odds Ratios (AOF)		
Independent Variables	AOR	[95% Conf Interval]	
**Total Children Ever Born**	1.140***	1.071	1.214
**Partner’s Age**	1.020*	1.003	1.037
**Place of Residence**			
Rural	1.000	-	-
Urban	0.615***	0.458	0.827
**Age Group**			
15–19	1.000	-	-
20–24	0.378**	0.177	0.805
25–29	0.326**	0.154	0.693
30–34	0.324**	0.149	0.708
35–39	0.281**	0.123	0.643
40–44	0.320**	0.132	0.774
45–49	0.333*	0.124	0.891
**Currently Working**			
No	1.000	-	-
Yes	0.781*	0.625	0.975
**Wealth Index**			
Poorest	1.000	-	-
Poorer	0.947	0.607	1.474
Middle	0.853	0.557	1.308
Richer	0.531**	0.332	0.850
Richest	0.422***	0.248	0.719
**Highest Level of Education**			
No Education	1.000	-	-
Primary	1.030	0.671	1.581
Secondary	0.784	0.491	1.252
Higher	0.697	0.332	1.466
**Religion**			
Catholic	1.0		
Protestant	1.232	0.913	1.661
Muslim	2.299	0.693	7.629
Other	2.138	0.780	5.860
**DHS survey year**			
2007	1.000	-	-
2013	0.893	0.626	1.273
2018	0.986	0.679	1.430

Note: * *p* <.05; ** *p* <.01; *** *p* <.001

## Discussion

The study findings suggest that unmet need for family planning among women living with HIV has hardly declined from 2007 to 2018. Essentially, it has not sustainably changed in a positive direction (i.e., reduction in unmet need) for slightly more than a decade– a period covered by this study. There is paucity of evidence showing trends in contraceptive use among women living with HIV in similar contexts making comparison with our study a difficult endeavour. Nonetheless, findings from some cross-sectional studies show similar levels reported in different survey points in our study. For example, a meta-analysis of unmet need for family planning among reproductive age women living with HIV in Ethiopia reported a pooled prevalence of 25% [26]. Similarly, an analysis of DHS data from twelve African countries with an HIV prevalence of more than 3%, including Zambia, showed an unmet need for family planning among women with HIV ranging from 9 to 23% [27].

With strategies on reducing unwanted pregnancies among women living with HIV indicated as an important prong in preventing vertical transmission of HIV for the past two decades, stagnation of unmet need for family planning in this population group reflects some shortcomings that warrant attention if benefits of prong 2 are to be optimally realised. In contrast, between 2010 and 2020, anti-retroviral treatment coverage among pregnant women living with HIV (prong 3) in Zambia markedly increased from 58% to > 95% [10] effectively narrowing the treatment gap.

As expected, an increasing trend in ART coverage among pregnant women living with HIV has corresponded with a declining trend in new HIV infections among children. Given prevailing evidence on significance of prong 2, it is highly probable that greater strides could have been made in reducing vertical transmission of HIV if unmet need for family planning reduced at a faster rate than current trends in Zambia.

Integrating family planning and ART services has emerged as one of the programming options to strengthen prong 2 for preventing vertical transmission of HIV in many high HIV burden countries including Zambia. Multiple modalities of integration such as; co-location of ART and family planning services, provider initiated family planning, capacity building of health workers for comprehensive SRH/HIV care [28–29] have been implemented with varying forms of design and results in different countries. In some cases, this has yielded positive results with evidence from Ethiopia [29], Zambia [30] and Kenya [31] suggesting increased contraceptive uptake and conversely reduction in unmet need for family planning where family planning and HIV services were integrated. Evidence of user satisfaction with services has also been demonstrated from systematic reviews [32–33]. However, these promising practices have either been implemented at a smaller scale or have not been fully integrated in the healthcare service delivery system resulting in limited nationwide benefits and impact. Furthermore, evidence is still sparse on long term benefits of some integration models [30]. Consequently, unmet need for family planning has hardly declined among women living with HIV as per our study findings.

Current discourse in preventing vertical transmission of HIV has rightly tended to focus on more targeted interventions informed by evidence on main sources of new HIV infections among children along the cascade of care. In Zambia, evidence shows that incident maternal HIV infections in the breastfeeding period, account for majority of vertical transmissions [10]. In this epidemiological context, strengthening family planning services through integration with HIV services in the postpartum period, serves two integral functions for prevention of vertical transmission of HIV.

Firstly, quality family planning counselling emphasizes dual protection from pregnancy and Sexually Transmitted Infections including HIV hence stands to directly contribute to reducing incident infections. Evidently, a study in Kenya found that integrating family planning and HIV services contributed to uptake of dual methods of protection from pregnancy and STI including HIV [31]. Secondly, for women who still acquire HIV during the breastfeeding period, it offers a choice at an early stage of HIV infection, to prevent unwanted pregnancies subsequently, thereby fully reaping the benefits of prong 2. Efficient programming approaches that identify women living with HIV most in need of family planning services in a timely manner (and at critical times) and offer opportunity to access multiple essential SRHR services are warranted.

Our analysis revealed that geographical residence (rural versus urban), age of a woman, socioeconomic status, number of children ever born to a woman (parity), employment status and partner’s age constituted factors significantly associated with unmet need for family planning. These findings are largely consistent with other studies [34–38] including a Zambian study which analysed unmet need for family planning using the 2018 DHS data [34]. From a review of literature, predictors of contraceptive uptake and unmet need for family planning are similar for HIV positive and HIV negative women.

Our study findings suggest that the more children a woman has the higher the chances they have an unmet need for family planning. This is in line with study findings from Zambia [34] Ethiopia [36–38] and Nigeria [39] including a multi-country study that analysed nineteen DHS survey data from Sub-Sahara Africa [40]. It is probable that with higher parity, women will have attained the desired number of children hence a greater need for limiting and in the context of limited access to contraceptive services, the unmet need is potentially high. Moreover, with higher parity there may be low risk perception pertaining to unwanted pregnancy due to perceived experience. Consequently, a programming implication entails the need for targeted family planning education and counselling for women with higher parity.

Similar to our findings, studies in Tigray [36] and Dire Dawa [38] in Ethiopia, Nigeria [34] and India [41] found that women living with HIV residing in rural areas were more likely to have unmet need for family planning than their counterparts from the urban areas. The probable explanation for this phenomenon is that women in rural areas have lower exposure to information and limited access to sexual and reproductive health services than their urban counterparts, as reported in surveys in Zambia and elsewhere [30, 34]. Furthermore, in the Zambia country context, women in rural areas are arguably impacted more than their urban counterparts by other barriers to uptake of family planning services such as some deeply engrained cultural practices that propagate hesitancy to contraceptives and those that affect the autonomy of women to make reproductive choices.

Women in high socioeconomic status had lesser odds of having unmet need for family planning than their counterparts in lower socioeconomic strata. This renders support to similar findings reported in other studies in Ghana [42], Pakistan [43] and Ethiopia [38]. The underlying pathway for this association is that women from wealthier households have greater ability to pay for direct and indirect costs associated with accessing modern contraceptive methods and are more likely to have better access to information on contraceptives and services than women from poorer households [38].

Consistent with other studies [27, 38, 40–41], our study found that women’s age is a significant predictor of unmet need for family planning. The pattern of association shows that women aged more than 20 years are less likely to have unmet need for family planning relative to younger women (below 20 years) (see Table 2). This phenomenon may be explained by the comparator age group of adolescence (15–19 years) granted established evidence of multiple barriers to accessing family planning services for adolescent girls. These include stigma and discrimination, unfriendly attitude of health providers, and inadequate information about contraceptives [44–48]. Indeed, additional evidence in the SRHR/HIV programming realm suggest that compared to older women, pregnant adolescent girls and young mothers aged 15 to 24 years are less likely to know their HIV status, to be on sustained HIV treatment and their children more likely to be infected by HIV [49]. Therefore, generating robust local evidence and targeted programming for this age group remains imperative across all essential interventions to eliminate vertical transmission of HIV. It is worthwhile to note that another finding of this study suggests that unmet need for family planning among women living with HIV increased with an increasing age of spouse, a phenomenon that could be related to partner’s decision-making dynamics. Programmatic implications related to engagement of spouses and ongoing education and counselling on reproductive health choices throughout the reproductive period are thus vital.

Our study also confirmed findings of earlier studies [43] where employed women had less odds of unmet need for family planning. The causal pathways for this phenomenon relate to the potential for employed women to be more empowered, have the financial capacity, better exposure to information to make reproductive health choices including uptake of family planning services.

While education has been shown to be a significant predictor of contraceptive uptake, and conversely of unmet need for family planning among women in other studies [38–39, 50–55], our findings did not demonstrate any association. It is however consistent with a study in Zambia that used the 2018 DHS data and involved all women (not restricted to those with an HIV positive result) [34]. This finding is arguably counter-intuitive and the mechanism for this phenomenon (lack of association with education) is unclear although it points to the importance of emphasizing family planning specific education and counselling irrespective of a woman’s education level.

Our study strengths relate to the use of data from DHS which are large population-based surveys considered robust enough to be nationally representative and frequently used to inform policy and practice. One salient limitation is that, while trends from cross-sectional survey points are useful and in our study context, most appropriate, they are not as robust as longitudinal studies for which temporal relationship could have been better ascertained. In the same manner, this repeated cross-sectional design entail using data from different individuals at different time points, rendering it likely that other factors not observable in the data may have some impact on some variables. However, our analysis controlled for different survey time points and further revealed that sociodemographic characteristics of participants from survey points were largely similar, the impact of this limitation is greatly minimized. Other limitations common to surveys including recall and reporting bias are also applicable to our study although we acknowledge the rigor of DHS in minimizing these biases.

## Conclusion

Our study findings show that unmet need for family planning among women living with HIV have stagnated for more than a decade despite a long-standing programming context and evidence on the need to improve contraceptive uptake for women living with HIV not desiring to have children as part of preventing vertical transmission of HIV and improving maternal health. Averting HIV infections in children preserves life and contributes to improving quality of life from its early stages. This is at the core of elimination of vertical transmission of HIV agenda [56]. Additionally, preventing one HIV infection in a child is averting lifetime costs of HIV treatment and associated healthcare costs. Investing in cost effective interventions such as prong 2 for prevention of vertical transmission of HIV is therefore a programmatic imperative given our findings that unmet need for family planning has hardly decreased over time.

The findings of this study point to various other implications for policy and practice. It is imperative that efforts are made for tailor-made interventions that are responsive to the reproductive health needs of women living with HIV who are most likely to have unmet need for family planning. From our study findings, greater programmatic attention is warranted for women with high parity, those residing in rural areas, unemployed women, adolescent girls and young women, and women from poor households.

The extent to which Zambia pursues the last mile towards path to elimination of vertical transmission of HIV partly depends on optimization of proven cost-effective interventions. Reducing unmet need for family planning among women living with HIV continues to hold promise in this regard.

## Data Availability

The datasets used and/or analysed during the current study are available from the corresponding author on reasonable request. The link for Demographic Health Surveys conducted in Zambia is provided here “https://dhsprogram.com/Countries/Country-Main.cfm?ctry_id=47&c=Zambia&Country=Zambia&cn=&r=1.

## Abbreviations

AIDS: Acquired Immuno-deficiecy Syndrome
ART: Anti-Retroviral Therapy
ARV: Anti-Retroviral drugs
DHS: Demographic and Health Survey
HIV: Human Immuno-deficiency Virus
PMTCT: Prevention of Mother To Child Transmission of HIV
RCS: Repeated Cross-sectional Survey
SGBV: Sexual and Gender Based Violence
SRHR: Sexual Reproductive Health and Rights
TaSP: Treatment as Prevention
WHO: World Health Organisation
ZDHS: Zambia Demographic and Health Survey

## References

[1] Sperling RS, Shapiro DE, Coombs RW, Todd JA, Herman SA, McSherry GD (1996). Maternal viral load, zidovudine treatment, and the risk of transmission of human immunodeficiency virus type from mother to infant. Pediatric AIDS clinical trials Group Protocol 076 Study Group. New Engl J Med.

[2] Guay LA, Musoke P, Fleming T, Bagenda D, Allen M, Nakabiito C (1999). Intrapartum and neonatal singledose nevirapine compared with zidovudine for prevention of mother-to-child transmission of HIV1 in Kampala, Uganda: HIVNET 012 randomised trial. Lancet.

[3] Shaffer N, Chuachoowong R, Mock PA, Bhadrakom C, Siriwasin W, Young NL et al. Short-course zidovudine for perinatal HIV-1 transmission in Bangkok, Thailand: a randomised controlled trial. Bangkok Collaborative Perinatal HIV Transmission Study Group. Lancet. 1999; 353(9155). 10.1016/s0140-6736(98)10411-7 10459957

[4] WHO. Prevention of Mother-To-Child Transmission (PMTCT). Briefing Note October 1st, 2007. Available from: https://www.who.int/hiv/pub/toolkits/PMTCT%20HIV%20Dept%20brief%20Oct%2007.pdf.

[5] Interagency Task Team (IATT) on Prevention of HIV Infection in Pregnant Women, Mothers and their Children. Guidance on global scale-up of the prevention of mother to child transmission of HIV: towards universal access for women, infants, and young children and eliminating HIV and AIDS among children. 2007. Available from: https://apps.who.int/iris/handle/10665/43728.

[6] Hairston AF, Bobrow EA, Pitter CS. (2012) Towards the Elimination of Pediatric HIV: Enhancing Maternal, Sexual, and Reproductive Health Services. International Journal of Maternal and Child Health and AIDS: 2012; 1(1): 6–16. 10.21106/ijma.13 PMC4948161 27621955

[7] WHO. Antiretroviral treatment as prevention (TasP) of HIV and TB.2012. Available from: https://apps.who.int/iris/bitstream/handle/10665/70904/WHO_HIV_2012.12_eng.pdf?sequence=1.

[8] UNAIDS. Invest in HIV Prevention: quarter for HIV prevention. Available from: https://www.unaids.org/sites/default/files/media_asset/JC2791_invest-in-HIV-prevention_en.pdf.

[9] Schwartländer B. (2011). Treatment 2.0: Translating concept into practice to overcome the HIV epidemic. Statement at UNAIDS and WHO seminar. Available from: https://www.unaids.org/en/resources/presscentre/featurestories/2011/march/20110324treatmentseminar.

[10] UNAIDS. 2021. Available from: https://aidsinfo.unaids.org/.

[11] UNAIDS. and PEPFAR. 2015 progress on the global plan towards the elimination of new HIV infections among children and keeping their mothers alive. 2015. Available from: https://www.unaids.org/sites/default/files/media_asset/JC2774_2015ProgressReport_GlobalPlan_en.pdf. 10.1097/QAI.0000000000001313 PMC5407381 28398999

[12] Reynolds HW, Janowitz B, Wilcher R, Cates W (2008). Contraception to prevent HIV-positive births: current contribution and potential cost savings in PEPFAR countries. BMJ Journals.

[13] Hladik W, Stover J, Esiru G, Harper M, Tappero J. The contribution of family planning towards the prevention of vertical HIV transmission in Uganda. PLoS ONE. 2009;4(11). 10.1371/journal.pone.0007691. 10.1371/journal.pone.0007691 PMC2766039 19888347

[14] Sherwood J, Lankiewicz E, Roose-Snyder B, Cooper B, Jones A, Honermann B (2021). The role of contraception in preventing HIV-positive births: global estimates and projections. BMC Public Health.

[15] Kassie MD, Habitu YA, Berasa SH. Unmet need for family planning and associated factors among women living with HIV in Gondar city, Northwest Ethiopia: cross-sectional study. Pan Afr Med J. 2021;38(22). 10.11604/pamj.2021.38.22.21431. 10.11604/pamj.2021.38.22.21431 PMC7955604 33777290

[16] Mkwashapi D, Renju J, Mahande M (2023). Unmet need for modern contraception by HIV status: findings from community—based studies implemented before and after earlier ART initiation program in rural Tanzania. Reprod Health.

[17] Rucinski KB, Powers KA, Schwartz SR, Pence W, Chi BH, Black V (2018). Longitudinal patterns of unmet need for contraception among women living with HIV on antiretroviral therapy in South Africa. PLoS ONE.

[18] Ministry of Health. National plan for the elimination of mother to child transmission of HIV and Syphilis. Lusaka, Zambia.

[19] WHO (2021). Global guidance on criteria and processes for validation: elimination of mother-to-child transmission of HIV, Syphilis and Hepatitis B virus.

[20] The DHS. Program. Zambia Demographic and Health Survey 2018.

[21] Hancock NL, Chibwesha CJ, Bosomprah S, Newman J, Mubiana-Mbewe M, Sitali ES, Chi BH (2016). Contraceptive use among HIV-infected women and men receiving antiretroviral therapy in Lusaka, Zambia: a cross-sectional survey. BMC Public Health.

[22] Matthew J, Lebo,Weber W. An effective approach to the repeated cross-sectional design. Am J Polit Sci. 59(1) 10.1111/ajps.12095.

[23] The DHS Program. Available from: http://dhsprogram.com/What-We-Do/Survey-Types/DHS.cfm.

[24] MEASURE DHS. Family planning and Reproductive health indicators database: Unmet need for family planning. Available from: https://www.measureevaluation.org/prh/rh_indicators/family-planning/fp/unmet-need-for-family-planning.

[25] Hosmer DW, Hosmer T, Cessie SLE, Lemeshow S. A comparison of goodness-of-fit tests for the logistic regression model. 1997; 1996:965–80. 10.1002/(sici)1097-0258(19970515)16:9<965::aid-sim509>3.0.co;2-o 9160492

[26] Kefale B, Adane B, Damtie Y, Arefaynie M, Yalew M, Andargie A (2021). Unmet need for family planning among reproductive-age women living with HIV in Ethiopia: a systematic review and meta-analysis. PLoS ONE.

[27] MacQuarrie KLD (2014). Unmet need for family planning among young women: levels and trends. DHS comparative reports No. 34. Rockville.

[28] Nkhoma L, Sitali DC, Zulu JM (2022). Integration of family planning into HIV services: a systematic review. Ann Med.

[29] Demissie DB, Mmusi-Phetoe R. Integration of family planning services with HIV treatment for women of reproductive age attending ART clinic in Oromia regional state, Ethiopia. Reprod Health. 2021;18. 10.1186/s12978-021-01157-0. 10.1186/s12978-021-01157-0 PMC8141201 34022885

[30] Hancock NL, Vwalika B, Sitali ES, Mbwili-Muleya C, Chi BH, Stuart GS (2015). Evaluation of service quality in family planning clinics in Lusaka. Zambia Contracept.

[31] Chen Y, Begnel E, Muthigani W, Achwoka D, Mcgrath CJ, Singa B, Gondi J, Ng’ang’a L, Langat A, John-Stewart G, Kinuthia J, Drake AL (2020). Higher contraceptive uptake in HIV treatment centers offering integrated family planning services: a national survey in Kenya. Contraception.

[32] Narasimhan M, Yeh PT, Haberlen S (2019). Integration of HIV testing services into family planning services: a systematic review. Reprod Health.

[33] Lindegren ML, Kennedy CE, Bain-Brickley D, Azman H, Creanga AA, Butler LM, Spaulding AB, Horvath T, Kennedy GE. Integration of HIV/AIDS services with maternal, neonatal and child health, nutrition, and family planning services. Cochrane Database Syst Reviews. 2012;9. 10.1002/14651858.CD010119. 10.1002/14651858.CD010119 PMC12551653 22972150

[34] Namukoko H, Likwa RN, Hamoonga TE, Phiri M (2022). Unmet need for family planning among married women in Zambia: lessons from the 2018 demographic and Health Survey. BMC Womens Health.

[35] Anik AI, Islam MR, Rahman MS (2022). Association between socioeconomic factors and unmet need for modern contraception among the young married women: a comparative study across the low- and lower-middle-income countries of Asia and Sub-saharan Africa. PLOS Glob Public Health.

[36] Melaku YA, Zeleke EG. Contraceptive utilization and associated factors among HIV positive women on chronic follow up care in Tigray region, Northern Ethiopia: A cross sectional study. PLoS One. 2014; 9 (4):1–10. 10.1371/journal.pone.0094682 PMID: 24743241. 10.1371/journal.pone.0094682 PMC3990566 24743241

[37] Tadele A, Abebaw D, Ali R (2019). Predictors of unmet need for family planning among all women of reproductive age in Ethiopia. Contracept Reprod Med.

[38] Dejene H, Abera M, Tadele A (2021). Unmet need for family planning and associated factors among married women attending antiretroviral treatment clinics in dire Dawa City, Eastern Ethiopia. PLoS ONE.

[39] Fagbamigbe AF, Afolabi RF, Idemudia ES. Demand and unmet needs of contraception among sexually active in-union women in Nigeria: Distribution, associated characteristics, barriers, and program implications. SAGE Open. 2018; 8(1). 10.1177/2158244017754023 PMID: 30221033.

[40] Ahinkorah BO, Ameyaw EK, Seidu AA, Ahinkorah (2020). Socio-economic and demographic predictors of unmet need for contraception among young women in sub-saharan Africa: evidence from cross-sectional surveys. Reprod Health.

[41] Koshewara P, Fuke RP (2019). Identifying the unmet need of contraception among HIV seropositive women attending antiretroviral treatment (ART) clinic in tertiary care centre. J Evid Based Med Healthc.

[42] Lauro D (2011). Abortion and contraceptive use in sub-saharan Africa: how women plan their families. Afr J Reprod Health/La Revue Africaine De La Santé Reproductive.

[43] Asif MF, Pervaiz Z (2019). Socio-demographic determinants of unmet need for family planning among married women in Pakistan. BMC Public Health.

[44] Juarez F, Gayet C, Mejia-Pailles G (2018). Factors associated with unmet need for contraception in Mexico: evidence from the National Survey of demo graphic dynamics 2014. BMC Public Health.

[45] Dejenu G, Ayichiluhm M, Abajobir AA (2013). Prevalence and associated factors of unmet need for family planning among married women in Enemay District, Northwest Ethiopia: a comparative cross-sectional study. J Com Munity Med Health Educ.

[46] Bain EL, Amu H, Tarkang EE (2021). Barriers and motivators of contraceptive use among young people in Sub-saharan Africa: a systematic review of qualitative studies. PLoS ONE.

[47] Ochako R, Mbondo M, Aloo S, Kaimenyi S, Thompson R, Temmerman M (2015). Barriers to modern contraceptive methods uptake among young women in Kenya: a qualitative study. BMC Public Health.

[48] Hokororo A, Kihunrwa AF, Kalluvya S, Changalucha J, Fitzgerald DW, Downs JA. Barriers to access reproductive health care for pregnant adolescent girls: a qualitative study in Tanzania. Actapaediatrica. 2015; 104 (12):1291–7. 10.1111/apa.12886 PMID: 25473729. 10.1111/apa.12886 PMC4456338 25473729

[49] UNICEF. (2020) Addressing the needs of adolescent and young mothers affected by HIV in Eastern and Southern Africa. Available from https://www.childrenandaids.org/sites/default/files/2020-10/UNICEF-ESA-Young-Mothers-HIV-Report-2020.pdf.

[50] Hailemariam A, Haddis F. Factors affecting unmet need for family planning in southern nations, nationalities and peoples region, Ethiopia. Ethiopian journal of health sciences. 2011; 21(2):77–90. 10.4314/ejhs.v21i2.69048 PMID: 22434988. 10.4314/ejhs.v21i2.69048 PMC3275860 22434988

[51] Ayele W, Tesfaye H, Gebreyes R, Gebreselassie T (2013). Trends and determinants of unmet need for family planning and programme options. Further analysis of the 2000, 2005, and 2011 demographic and health surveys.

[52] Wafula SW (2015). Regional differences in unmet need for contraception in Kenya: insights from survey data. BMC Women’s Health.

[53] Teshale AB. Factors associated with unmet need for family planning in Sub-saharan Africa: a multilevel multinomial logistic regression analysis. PLoS ONE. 2022;17(2). 10.1371/journal.pone.0263885. 10.1371/journal.pone.0263885 PMC8830726 35143584

[54] Wulifan JK, BrennerS, Jahn A, et al. A scoping review on determinants of unmet need for family planning among women of reproductive age in low and middle income countries. BMC Womens Health. 2015;16(2). 10.1186/s12905-015-0281-3. 10.1186/s12905-015-0281-3 PMC4714507 26772591

[55] Cleland J, Harbison S, Shah IH. Unmet need for contraception: issues and challenges. Stud Fam Plann. 2014;45(2):105–22. 10.1111/j.1728-4465.2014.00380.x 24931071

[56] UNAIDS. The Global plan to eliminate new HIV infections among children and keeping their mothers alive. 2011; Available from: https://www.unaids.org/sites/default/files/media_asset/JC2774_2015ProgressReport_GlobalPlan_en.pdf.

